# Antiangiogenic therapy exerts antitumor effects by altering the tumor microenvironment: bibliometric analysis

**DOI:** 10.3389/fimmu.2024.1460533

**Published:** 2024-12-03

**Authors:** Yuan Kang, Yixin Kang, Dianbao Zhang, Jun Yao

**Affiliations:** The First Affiliated Hospital, and College of Clinical Medicine of Henan University of Science and Technology, Luoyang, China

**Keywords:** antiangiogenic, angiogenesis, the tumor microenvironment, bibliometric, therapy

## Abstract

**Background:**

Antiangiogenic therapy can alter the tumor microenvironment (TME) and thus exert anti-tumor effects, and has the potential to increase the efficacy of conventional therapy and immunotherapy. The aim of this study was to examine current research hotspots and collaborative networks on the relationship between previous antiangiogenic therapies and the TME through bibliometric analysis.

**Method:**

From the Web of Science Core Collection database, all publications from inception through December 2023 were downloaded. In-depth analysis was performed by Bibliometrix packages in R. Keywords and collaborative networks were analyzed using VOSviewers and Citespace.

**Result:**

We obtained a total of 9027 publications. They come from 27 countries and were published in 1387 journals, with a total of 39,604 authors in the studied area. The number of publications increases dramatically from 2014 to 2023, accounting for 73.87% (6668/9027) of all publications. China and CANCERS have the highest number of publications on this topic and CANCER RESEACH is the most influential. In the last decade (2013- 2023), research has gradually shifted from studying the role of vascular endothelial growth factor in the TME to examining how antivascular therapy can contribute to the progression of cancer treatment. Furthermore, nanoparticle-based drug delivery systems and immunotherapy have been widely explored in the past five years. The findings of this study will help scientists to explore this promising field in depth by providing insight into the relationship between antiangiogenic therapy and the TME.

**Conclusion:**

The relationship between the antiangiogenic therapy and the TME has been developing rapidly, but cooperation between different institutions and countries is still limited. Researchers can use this study to identify hotspots and develop trends for related research, thereby facilitating the development and cooperative exchange in this field, as well as to suggest potential future research directions.

## Introduction

Angiogenesis, the formation of new blood vessels, is a dynamic process regulated by a variety of pro-angiogenic and antiangiogenic molecules, which plays a crucial role in tumor growth, invasion, and metastasis (1, 2), and may contribute to the progression of a variety of malignant tumors, such as breast cancer (BC), colorectal cancer (CRC), and non-small-cell lung cancer (NSCLC), and so on (3, 4). With the development of molecular and cellular biology, the role of various biomolecules such as growth factors, chemokines, and adhesion factors in tumor angiogenesis has been gradually elucidated thus promoting antiangiogenic therapy as a promising strategy for antitumor therapy (5). As an emerging therapeutic tool, antiangiogenic therapy limits tumor growth and spread by normalizing tumor vasculature, increasing drug concentration in tissues, reducing microenvironmental hypoxia, and limiting distant invasion and metastasis of tumors (6).Despite the multitude of regulators involved in tumor angiogenesis, the most widely used antiangiogenic agents include monoclonal antibodies against the epidermal growth factor (EGF) (7),vascular endothelial growth factor (VEGF) pathway and tyrosine kinase inhibitors (TKIs) due to the different dominance of signaling pathways (8). TME is the local environment surrounding tumor cells, which consists of many types of cells, extracellular matrix (ECM), secreted factors, and signaling molecules, especially the synthesis and remodeling of the ECM (9),which has been shown to have a pro-tumorigenic effect. TME plays a crucial role in tumorigenesis, progression, metastasis, and response to therapy, and is therefore expected to be a therapeutic target for tumor intervention (10).

Antiangiogenic therapy cut off the nutrient and oxygen supply to the tumor by inhibiting multi-targeted angiogenic factors in the TME such as VEGF, thus preventing tumor angiogenesis (11); at the same time, they can reverse the state of immunosuppression in the TME, and promote the infiltration and activation of immune cells, thus enhancing the immune response to the tumor (12). Bibliometrics is an interdisciplinary discipline that applies mathematical and statistical methods to quantitatively analyze the literature and scholarly outputs as an analytical tool not only to qualitatively and quantitatively analyze the contributions and collaborations of authors, organizations, countries, and journals in a chosen field of study, but also to assess the development of and emerging trends in scholarly research (13), which are not possible with traditional meta-analyses and retrospective studies. This study uses the leading bibliometric software packages CiteSpace and VOSviewer to describe emerging trends in the relationship between antiangiogenic therapy and the TME from the following three perspectives (14): (1) Quantified information on individual impact and collaboration in antiangiogenic therapy- TME based on annual publications, country/institution, journals, authors, and co-cited authors. (2)Assessed the knowledge base of antiangiogenic therapy- TME by identifying the most cited articles through the analysis of the co-cited literature. (3) Discovered the evolution of the knowledge structure and hotspots in this field of research by utilizing keywords and the analysis of the co-cited literature (15, 16).

## Methods

Bibliometric information were searched from Web of Science of Core Collections (WoSCC) on 2024/5/23 with the following keyword query. (TS=Angiogenesis inhibitor OR TS= antiangiogenic AND TS= Tumor microenvironment OR TS= Cancer Microenvironment OR TS= Microenvironment, Cancer) The resulting records contained 1,758 publications, including 7,105 articles, 3,603 reviews, 280 book chapters, 149 proceedings, 68 early accesses, 64 editorials, 36 abstracts, one letter, and one news articles. We selected information of the bibliometric data including keywords, journal, authors, institution, country, subject category, number of citations, year of publication, and reference records. Mainstream bibliometric software includes VOSviewer (version 1.6.20), CiteSpace (V.6.3.R1), and so on was used to analyze the Co-occurrence of keywords, co-authorship, and co-citation analysis between countries/regions.

## Results

### Overview of annual publications

**Figure 1:**
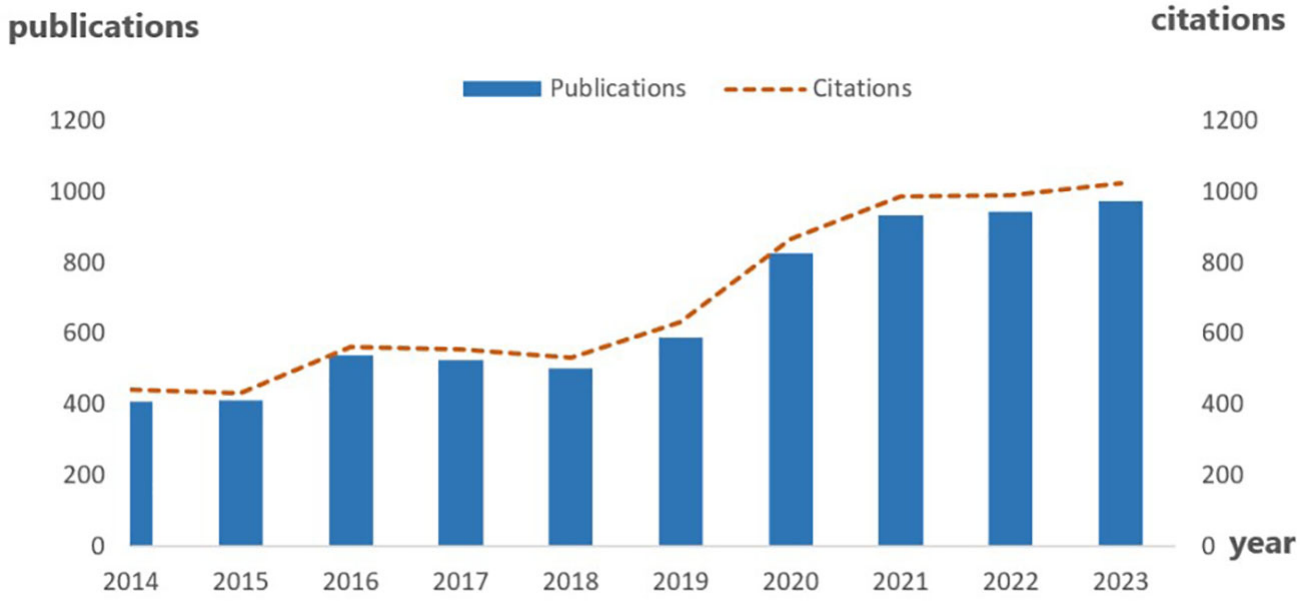
Number of publications and citations per year on tumor antiangiogenic therapy and TME.

Over the past decade, there has been a consistent rise in the annual output of publications and the number of citations in the field of tumor antiangiogenic therapy. The year 2023 marked a significant milestone with 1024 articles published and 926 citations recorded (as shown in **Figure 1**). Notably, the most substantial growth in citations occurred during two specific periods: from 2015 to 2016 and from 2018 to 2021. This upward trend persists and is indicative of the substantial research interest that anti-angiogenic therapy has garnered over the last ten years. It suggests that pivotal advancements or breakthroughs during those periods have stimulated further academic discourse and subsequent investigative efforts in the domain of anti-angiogenic treatment.

### The most-productive institutions and countries

**Figure 2:**
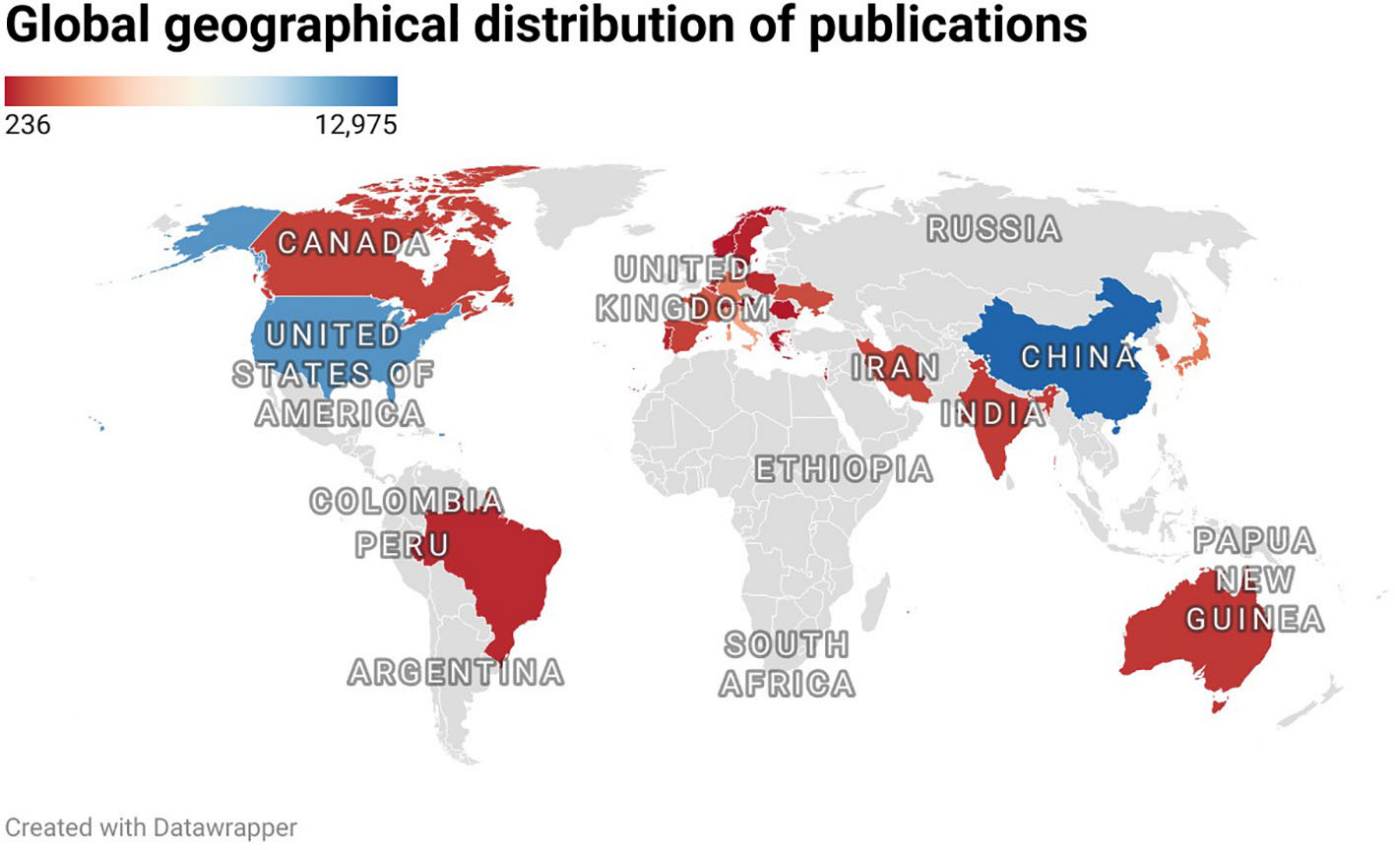
Geographic distribution of publications around the world.

**Figure 3:**
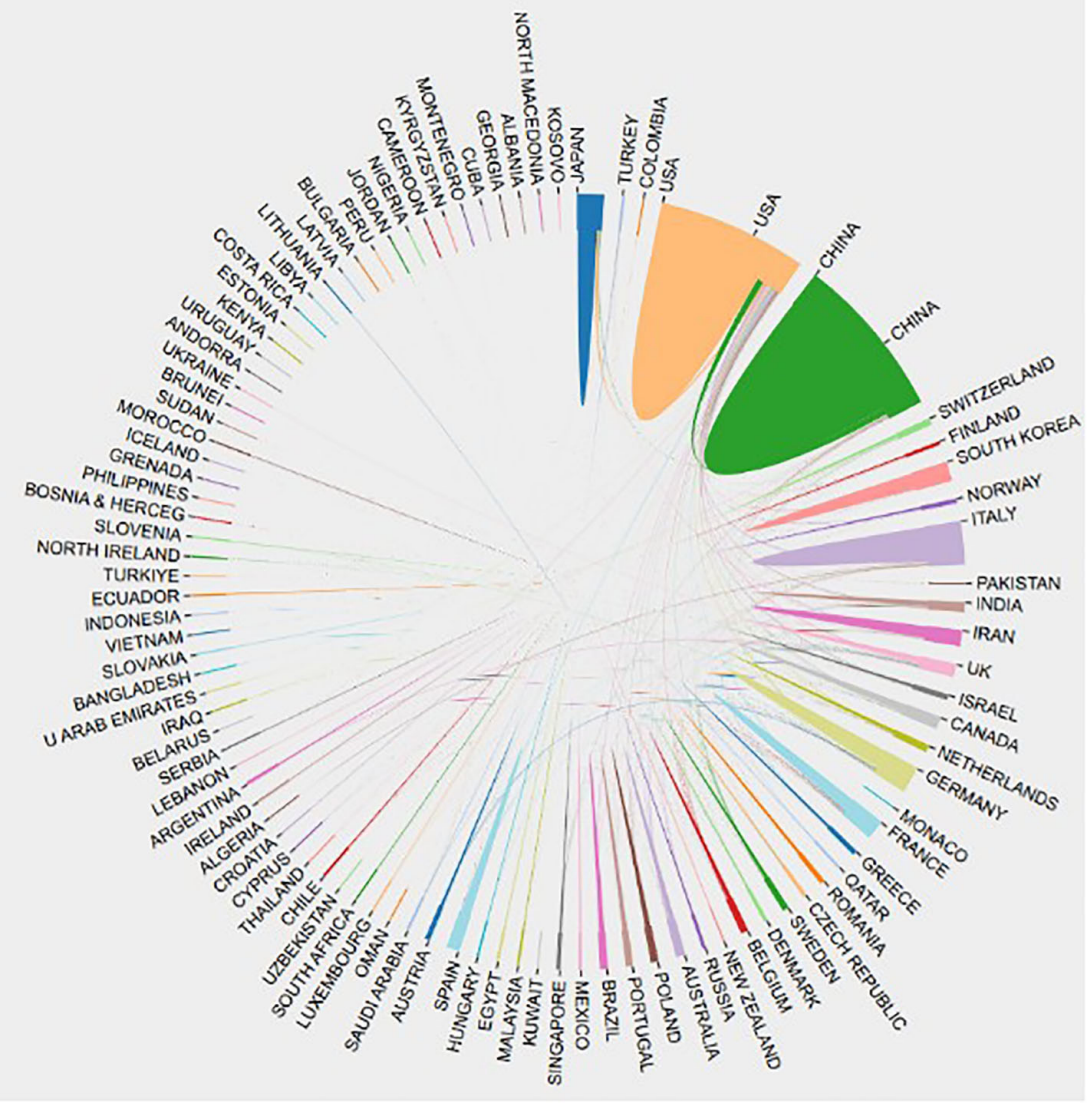
Visualization map of collaborations between countries/regions.

Based on the collected publications collected (**Table 1**), China made the most contribution to the publication of articles(2,488,27.6%), followed by the USA(2.230,24.7%) and Italy(643,7.1%). In addition, publications from the United States were cited first among all countries(222,188 times), followed by China (81,481 times) and Switzerland (53,524 times).Most publications were published by authors from Asia and North America, with Asia producing 1.13 times as many publications as North America (**Figure 2**). According to the international cooperation visualization map (**Figure 3**), China and the United States have the closest cooperative relationship, followed by the United States and Italy, the United States and Germany, the United States and Japan, the United States and the United Kingdom, while the relationships between other countries were relatively scattered. Among major research institutions, FUDAN UNIV topped the list with the highest publications(467 papers), while SICHUAN UNIV has 434 papers (**Table 2**). It was followed by UNIV TEXAS MD ANDERSON CANC CTR, SUN YAT SEN UNIV, and SHANGHAI JIAO TONG UNIV, all of these have published more than 350 articles, respectively. Geographically, most of the leading institutions come from China, the United States and Iran, further demonstrating the robust research of the relationship of antivascular therapy and TME in these countries. Geographically, most articles originate from China, the United States and Italy, which now further indicates the maturity and robustness of the research in the above countries.

**Table 1:** Top 10 most contributing countries in the field of the antiangiogenic-TME based on number of publications.

Rank	Country	Publication	Percentage	Total citation
1	CHINA	12975	0.348	81481
2	USA	11501	0.311	222188
3	ITALY	3115	0.089	476832
4	JAPAN	2009	0.059	20448
5	GERMANY	1872	0.046	16336
6	SOUTH KOREA	1386	0.036	10171
7	FRANCE	1345	0.032	11742
8	UK	1069	0.030	16661
9	IRAN	985	0.026	5043
10	CANADA	867	0.023	9162

**Table 2:** Ranking of top 10 institutions involved in the relationship between antiangiogenic therapy and the tumor microenvironment.

Rank	Institutions	Country	Number of articles	Percentage (%)
1	Fudan University	CHINA	467	1.01
2	Sichuan University	CHINA	434	0.94
3	The University of Texas MD Anderson Cancer Center	USA	432	0.93
4	Sun Yat-sen University	CHINA	396	0.85
5	Shanghai Jiao Tong University	CHINA	350	0.75
6	Harvard University	USA	345	0.74
7	Zhejiang University	CHINA	316	0.68
8	Nanjing Medical University	CHINA	310	0.67
9	Central South University	CHINA	301	0.65
10	Zhengzhou University	CHINA	271	0.58

### Leading publication sources

**Figure 4:**
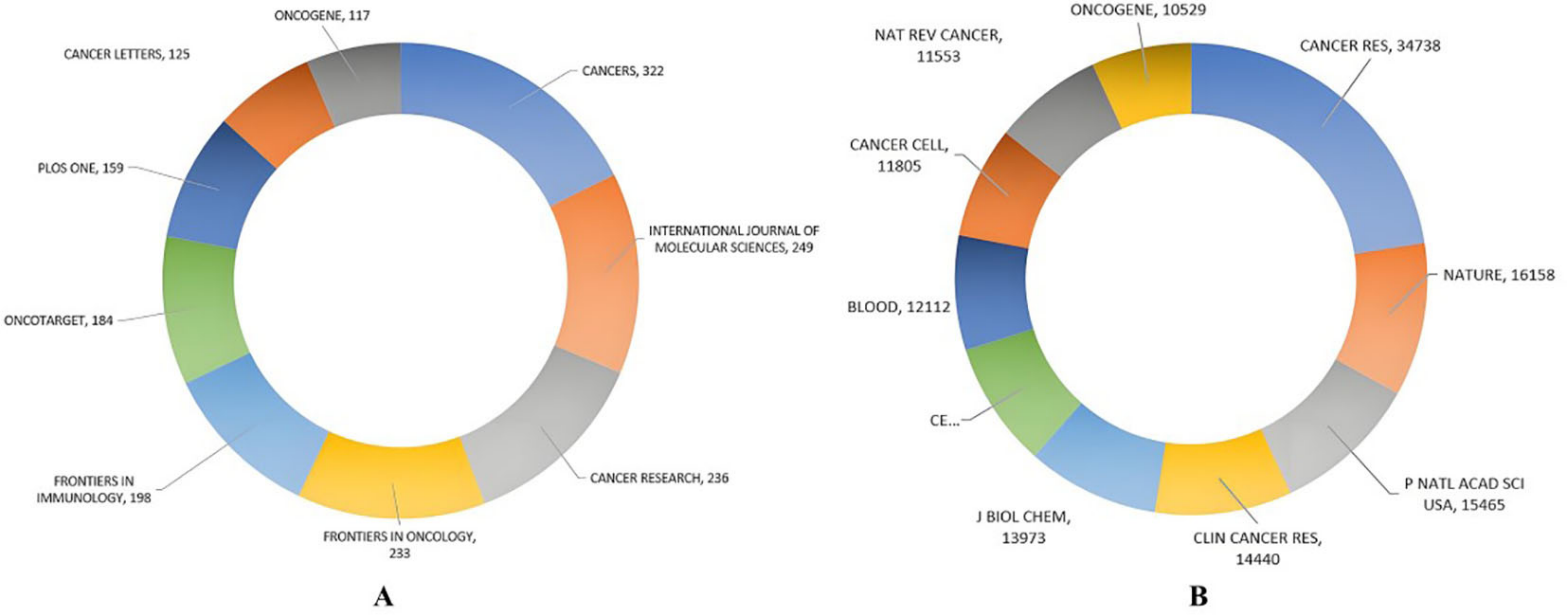
The top-ten **(A)** publishing journals and **(B)** cited journals in the field of the relationship between antiangiogenic therapy and the TME.

**Figure 5:**
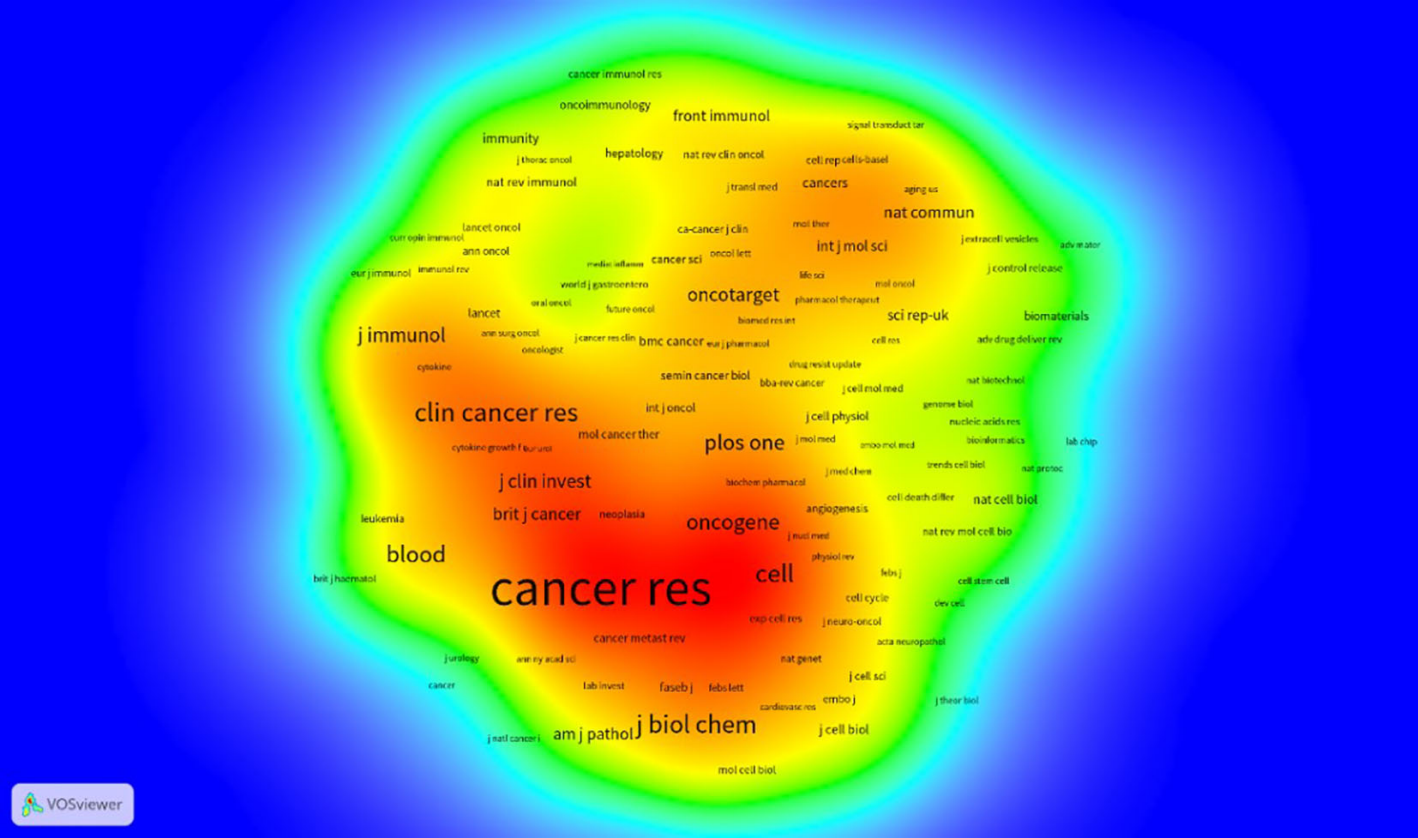
Density map of journals based on cocitations.

The ten leading journals published a total of 1932 articles on the relationship between antivascular therapy and TME. Among them, CANCERS publishes the most articles, with 322 articles (**Figure 4**), and The International Journal of Molecular Sciences is ranked second with 249 entries, but whether the citation ranking is consistent with the publication ranking. As the most-influential journal, CANCER RES has published 236 articles and has been cited 34,738 times, whereas NATURE ranked the second with 16,158 total citations. In the journal density map derived from the co-citation network (**Figure 5**), the influence of journals is discernible through the color intensity—red zones denote journals with a high citation frequency, underscoring their significance within the academic sphere, whereas blue zones signify journals that receive comparatively fewer citations. This visual representation allows us to distinctly identify that journals such as CANCER RESEARCH, ONCOGENE, CELL, CLINICAL CANCER RESEARCH, among others, are the most authoritative and influential publication outlets in the scholarly domain dedicated to the study of the interplay between anti-angiogenic therapy and the TME. These journals not only disseminate a plethora of high-caliber research articles but also enjoy extensive readership and citation, thereby consolidating their preeminent status in their respective fields.

### Leading publication authors

During the decade spanning from 2013 to 2023, a notable figure emerged in the field of research concerning the interplay between antivascular therapy and the TME (**Table 3**): Ribatti Domenico. With an impressive output of 57 scholarly articles dedicated to this subject, Ribatti Domenico has garnered significant attention, as evidenced by the 2,147 citations his work has received. However, when it comes to the sheer volume of citations as a measure of influence, Jain Rakesh takes the lead with a staggering 8,393 citations. This figure underscores the profound impact of his contributions to the scientific community. Sood Anil follows closely behind, with an equally commendable 2,356 citations, indicating a substantial body of work that has been widely recognized and referenced. These distinguished researchers hail from prestigious institutions known for their cutting-edge research and academic excellence. Ribatti Domenico is affiliated with the University of Bari Aldo Moro, a renowned center of learning in Italy. Jain Rakesh, on the other hand, is associated with Harvard University, one of the most esteemed universities globally, known for its rigorous academic standards and groundbreaking research. Lastly, Sood Anil is a part of The University of Texas MD Anderson Cancer Center, which is a leading institution in cancer research and treatment.

**Table 3:** Top productive authors in the field of the relationship between antiangiogenic therapy and the tumor microenvironment sorted by total publication numbers.

Rank	Author	Institutions	Country	Publications	Total citations
1	Ribatti Domenico	University of Bari Aldo Moro	Italy	57	2147
2	Yang Yang	Sichuan University	China	29	1354
3	Liu Yang	Fudan University		27	1895
4	Takabe Kazuaki	Roswell Park Comprehensive Cancer Center	USA	26	716
5	Jain Rakesh	Harvard University	USA	26	8393
6	Sood Anil	The University of Texas MD Anderson Cancer Center	USA	25	2356
7	Li Yan	Jinzhou Medical University	China	25	943
8	Vacca Angelo	University of Bari Aldo Moro	Italy	23	998
9	Wang Wei	China Pharmaceutical University	China	23	1288
10	Tamma Roberto	University of Bari Aldo Moro	Italy	22	563

### The most-cited publications

Citation analysis is considered to be an important method to evaluate the influence of articles. In addition, the analysis of cited articles is beneficial to determine the research hotspots. Herein, the 10 top-cited articles were selected according to the average number of citations per year (**Table 4**). The most frequently cited articles are published on CELL by HANAHAN D et al., and are cited an average of 3240.8 times a year. This article reviewed several therapies, including antiangiogenic therapy, that affect tumor treatment by creating tumor microremission (17). The second top-influential article, published by COUSSENS LM et al., is cited an yearly average of 495.48 times. This publication revealed that antivascular therapy plays a key role in regulating TME and illustrates the direct and important relationship between inflammation and tumor (18). Therefore, the effect of antiangiogenic therapy on TME is a very attractive and research topic.

**Table 4:** The 10 most cited publications based on average citation per year.

Rank	Paper	Article	DOI	Total Citations	TC per Year	Normalized TC
1	HANAHAN D, 2011, CELL	Hallmarks of cancer: the next generation	10.1016/j.cell.2011.02.013	45371	3,240.79	175.65
2	COUSSENS LM, 2002, NATURE	Inflammation and cancer	10.1038/nature01322	11396	495.48	36.45
3	MANTOVANI A, 2008, NATURE	Cancer-related inflammation	10.1038/nature07205	8334	490.24	51.37
4	CARMELIET P, 2000, NATURE	Angiogenesis in cancer and other diseases	10.1038/35025220	7168	286.72	17.05
5	KESSENBROCK K, 2010, CELL	Matrix metalloproteinases: regulators of the tumor microenvironment	10.1016/j.cell.2010.03.015	3716	247.73	29.18
6	NOY R, 2014, IMMUNITY	Tumor-associated macrophages: from mechanisms to therapy	10.1016/j.immuni.2014.06.010	2754	250.36	35.37
7	BERGERS G, 2003, NAT REV CANCER	Tumorigenesis and the angiogenic switch	10.1038/nrc1093	2736	124.36	18.21
8	LU P, 2012, J CELL BIOL	The extracellular matrix: a dynamic niche in cancer progression	10.1083/jcb.201102147	2205	169.62	27.27
9	COLOTTA F, 2009, CARCINOGENESIS	Cancer-related inflammation, the seventh hallmark of cancer: links to genetic instability	10.1093/carcin/bgp127	2091	130.69	19.23
10	CONDEELIS J, 2006, CELL	Macrophages: obligate partners for tumor cell migration, invasion, and metastasis	10.1016/j.cell.2006.01.007	2077	109.32	12.94

### Co-citation analysis of references

**Figure 6:**
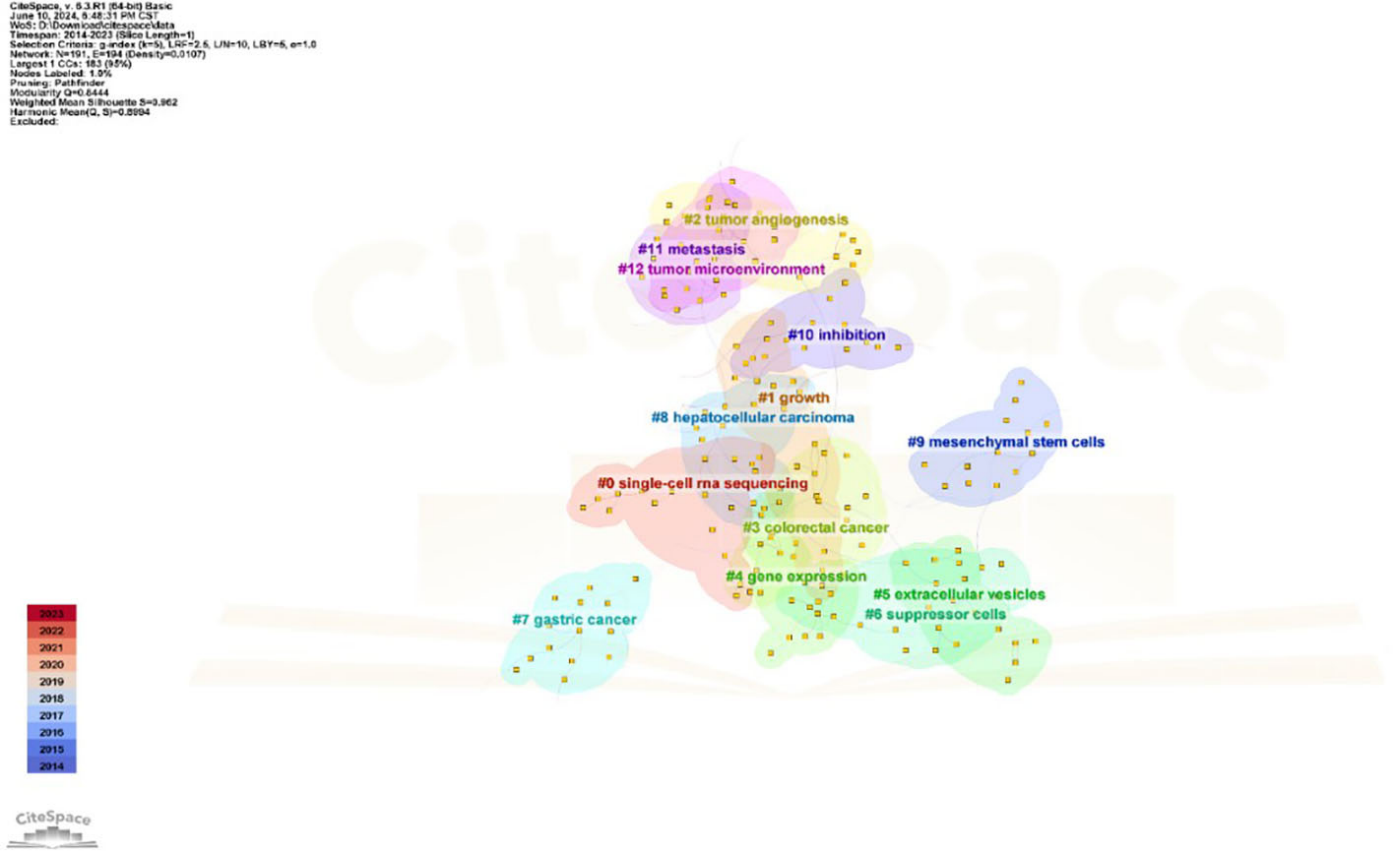
Clustering analysis of the relationship between antiangiogenic therapy and the TME co-citation network.

**Figure 7:**
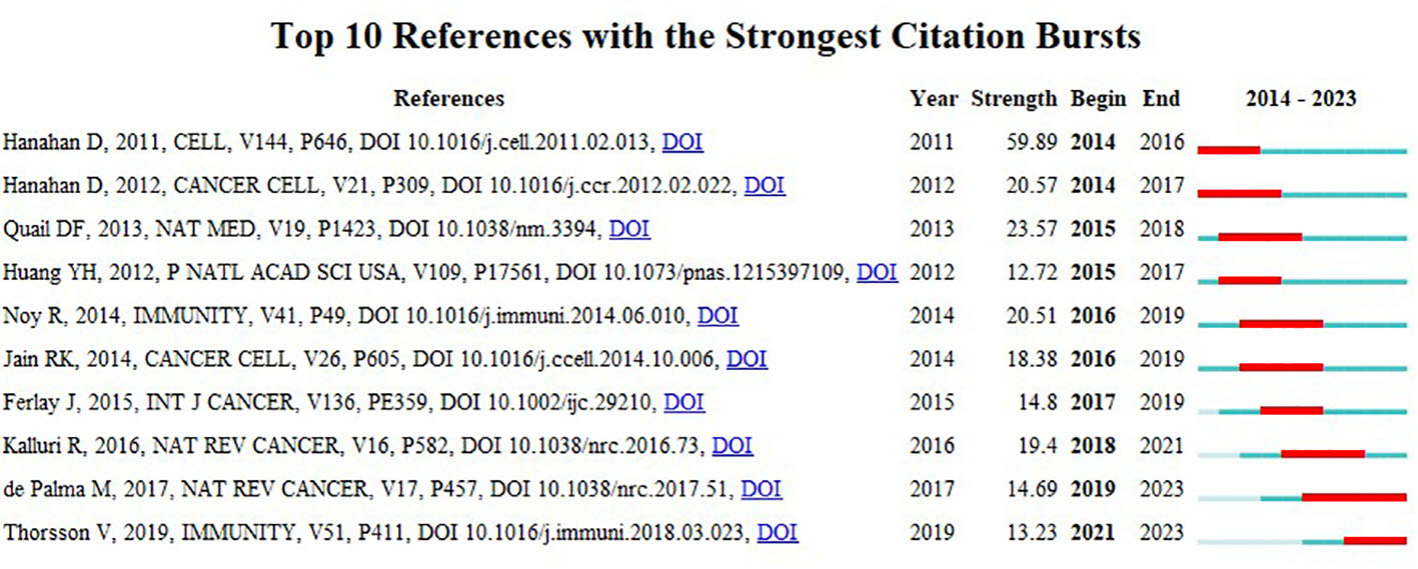
Top ten co-citations with the strongest bursts of citations.

Having two other references in the same publication both cited is referred to as having a co-citation relationship, as shown in **Figure 6**. The co-citation network is obtained by clustering operation on the basis of the cited articles, and using the latent semantic indexing (LSI) algorithm to extract noun terms from the keywords, which are divided into 13 clusters, with a clustering module value of 0.844, and a cluster mean contour value of 0.962, and S > 0.1 indicates a highly significant clustering structure and a convincing clustering effect. The antitumor effect demonstrated by antivascular therapy in various types of tumors is characterized by a clear clustering, whereby different tumors exhibit similar biological changes in response to antivascular therapy. This clustering may be attributed to a number of key factors, the first of which is the inherent limitations of antivascular drugs. For example, the accumulation of tumor stem cells represents a significant challenge. Tumor stem cells are characterized by self-renewal capacity and multidirectional differentiation potential, and they play a pivotal role in tumor origin, progression, metastasis and recurrence (19, 20). As antivascular therapy may not entirely eliminate these stem cells, resulting in their accumulation over the course of treatment, this may potentially diminish the therapeutic efficacy. Secondly, the issue of resistance to antivascular drugs must be considered. Tumor cells may gradually develop resistance to therapeutic drugs, which not only limits the long-term efficacy of the drugs but may also lead to recurrence after tumor treatment (21). Furthermore, the clustering effect of antivascular therapy may also be related to certain commonalities in the tumor microenvironment, such as the presence of immunosuppressive cells, which may influence the therapeutic effect in a similar way in different tumors (22). **Figure 7** and **Table 5** shows the top ten citation bursts, which are defined as articles that received a spike in citations in a given time period, indicating their rapid dissemination and widespread recognition in the research field (23). This figure shows that the main themes of the strong outburst were the link between the TME and tumors and how antivascular treatment of tumors can increase therapeutic efficacy (24). The highest citation burst intensity (59.89) was published in CELL by Hanahan D et al., and this publication with an overview of how TME will increasingly impact the development of new approaches to treating human cancer s (17).

**Table 5:** Top 10 reference with the strongest citation bursts.

Papers	Reference	Year	Strength
Hallmarks of cancer: the next generation	Hanahan D, CELL, V144, P646, DOI 10.1016/j.Cell.2011.02.013, DOI	2011	59.89
Accessories to the crime: functions of cells recruited to the tumor microenvironment	Hanahan D, CANCER CELL, V21, P309, DOI 10.1016/j.ccr.2012.02.022, DOI	2012	20.57
Microenvironmental regulation of tumor progression and metastasis	Quail DF, NAT MED, V19, P1423, DOI 10.1038/nm.3394, DOI	2013	23.57
Vascular normalizing doses of antiangiogenic treatment reprogram the immunosuppressive tumor microenvironment and enhance immunotherapy	Huang YH, P NATL ACAD SCI USA, V109, P17561, DOI 10.1073/pnas. 1215397109, DOI	2012	12.72
Tumor-associated macrophages: from mechanisms to therapy	Noy R, IMMUNITY, V41, P49, DOI 10.1016/j.immuni. 2014.06.010, DOI	2014	20.51
Antiangiogenesis strategies revisited: from starving tumors to alleviating hypoxia	Jain RK, CANCER CELL, V26, P605, DOI 10.1016/j.ccell.2014.10.006, DOI	2014	18.38
Cancer incidence and mortality worldwide: sources, methods and major patterns in GLOBOCAN 2012	Ferlay J, INT J CANCER, V136, PE359, DOI 10.1002/ijc.29210, DOI	2015	14.8
The biology and function of fibroblasts in cancer	Kalluri R, NAT REV CANCER, V16, P582, DOI 10.1038/nrc.2016.73, DOI	2016	19.4
Microenvironmental regulation of tumor angiogenesis	de Palma M, NAT REV CANCER, V17, P457, DOI 10.1038/nrc.2017.51, DOI	2017	14.69
The Immune Landscape of Cancer	Thorsson V, IMMUNITY, V51, P411, DOI 10.1016/j.immuni.2018.03.023, DOI	2019	13.23

### Analysis of keyword co-occurrence

**Figure 8:**
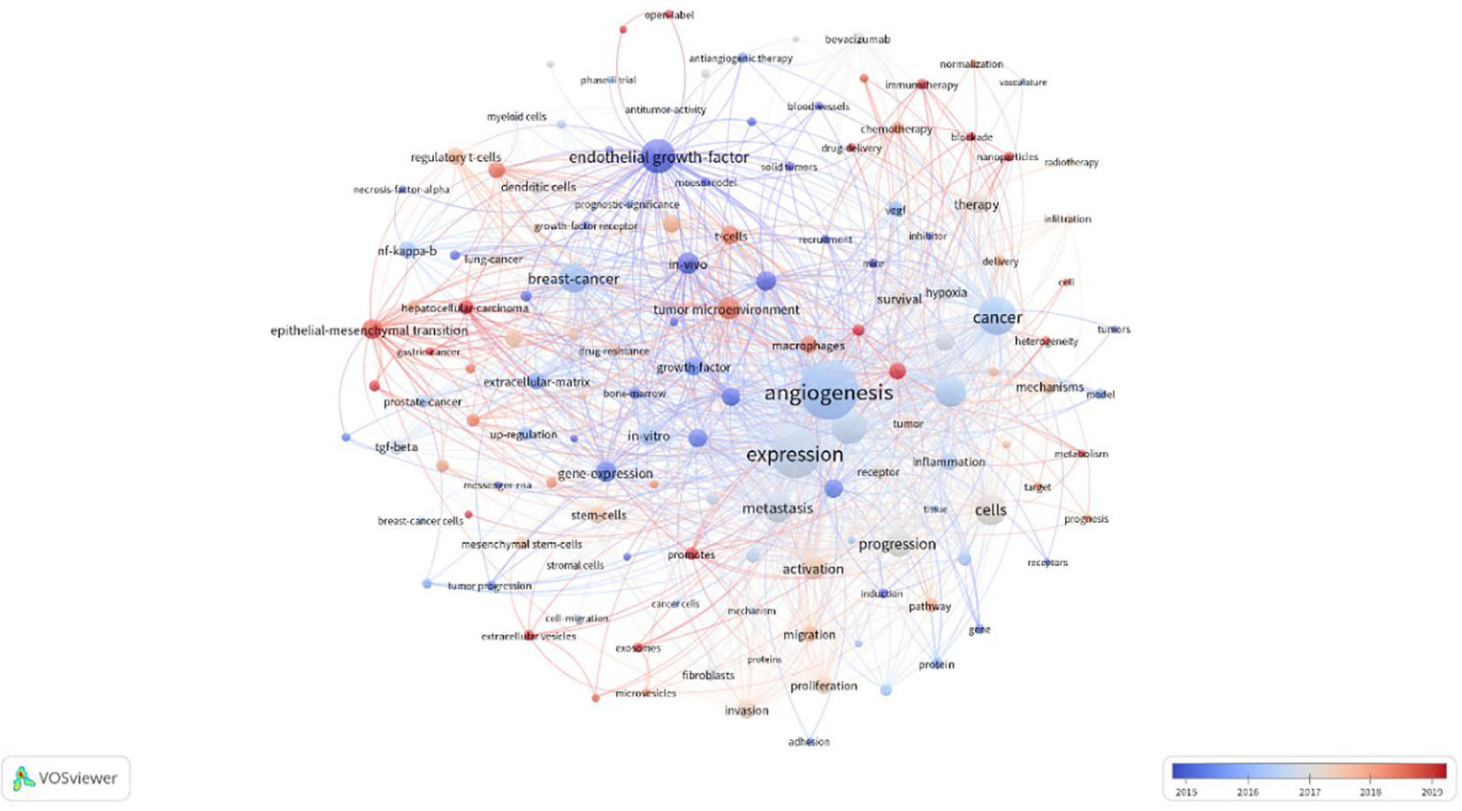
A chronological overview of keywords based on the average publication year.

Keywords are the framework of the article, which represent the theme of the research, is an extremely important part of the article. In this study, 92keywords from 21,105 with a highly frequency of 150 and above were analyzed by using software(VOSviewer). In the past 10 years, the top-ten keywords were ‘angiogenesis’,’expression,’cancer’,’growth’,’cells’,’endothelial growth-factor’, ‘microenvironment’, ‘breast-cancer’, ‘metastasis’ and ‘progression’. The node size of each keyword is proportional to the frequency of keyword occurrence, and the larger the node, the higher the frequency of keyword occurrence. The evolution of keywords over time is shown in **Figure 8**. Keywords colored in white or blue reflect an earlier period of prominence in publication, suggesting their foundational role in the field’s literature. Orange-shaded keywords indicate their usage throughout the timeline of this study. In contrast, those in red denote emerging research hotspots, signaling areas of growing interest and recent scholarly focus. Take ‘angiogenesis,’ for instance, a keyword that has been under scrutiny since the field’s nascent stages, alongside concurrent examinations of the expression of related receptors and the intricate dynamics of tumor metabolism. The investigation into the role of anti-angiogenic therapies in cancer treatment, particularly in their influence on the TME, has seen a discernible evolution in research focus. It has pivoted from an emphasis on the vascular endothelial growth factor’s part in the TME to a broader exploration of how anti-angiogenic therapies contribute significantly to oncology treatment strategies. Moreover, innovative topics such as nanoparticle-based drug delivery systems, exosomes, and immunotherapy have not only been widely utilized in the domain of cancer anti-angiogenic therapies over the past half-decade but also persist in capturing the research community’s attention. This visual proximity of nodes on the bibliometric map mirrors the conceptual closeness of the corresponding keywords, underscoring the interconnectedness and relevance of these themes within the current research discourse.

**Figure 9:**
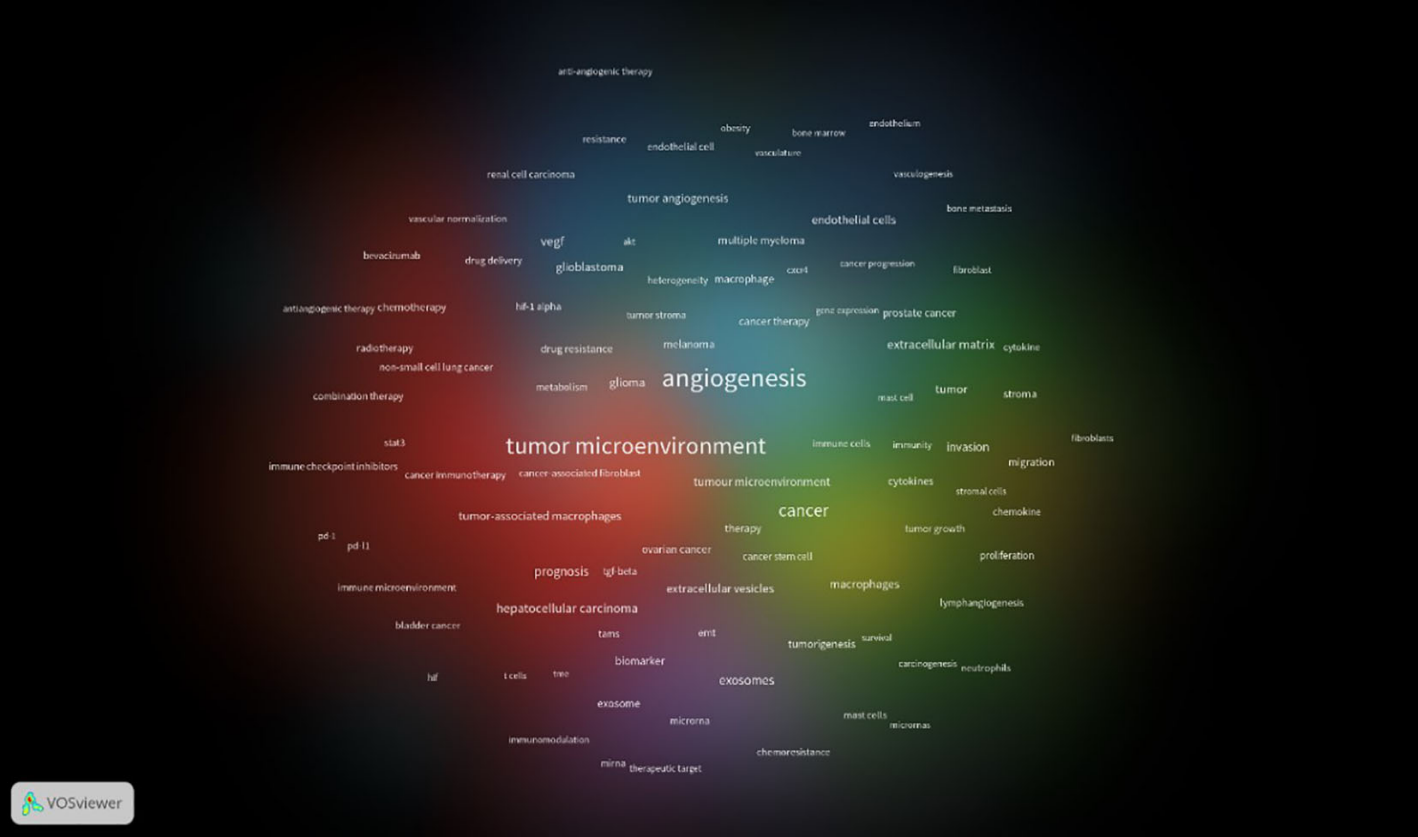
Map of keyword clusters based on research field of the relationship between antiangiogenic therapy and the TME.

To enhance clarity and visual representation in understanding the thematic interconnections and scholarly emphases within a particular academic sphere, we have categorized the 91 core keywords into four distinct clusters, as illustrated in **Figure 9**. Cluster 1, demarcated by the color red, is the most expansive and comprises 42 items, predominantly linked to the interface between the TME and cancer. It encompasses pivotal keywords such as ‘stromal cells,’ ‘suppressor cells,’ ‘endothelial cells,’ ‘exosomes,’ ‘cancer,’ ‘growth factor,’ and ‘metastasis.’ Cluster 2, highlighted in yellow, contains 26 items that concentrate on the microenvironment’s influence within the tumor’s progression, with keywords like ‘activation,’ ‘apoptosis,’ ‘fibroblasts,’ ‘growth,’ ‘invasion,’ and ‘polarization,’ which underscore the dynamic processes at play in tumor development. Cluster 3, depicted in blue, consists of 22 items that spotlight the therapeutic impact of anti-angiogenic treatments on the TME and address the challenges of potential resistance. It includes terms such as ‘anti-angiogenic therapies,’ ‘angiogenesis,’ ‘bevacizumab,’ ‘hypoxia,’ ‘microenvironment,’ and ‘drug resistance,’ which reflect the ongoing exploration of treatment mechanisms and the complexities of therapeutic resistance. Lastly, Cluster 4, represented in purple, is intricately associated with the role of the TME in melanoma, indicating a specialized research focus within the broader context of oncology. This systematic clustering not only lays bare the multifaceted dimensions of research within the TME and oncology but also accentuates the evolving research trends and potential knowledge gaps that may steer future investigative endeavors in the field.

## Discussion

Over the past decade, anti-angiogenic therapy has continued to attract the interest of researchers around the world, and how it changes TME is an important strategy in anti-tumor and anti-tumor therapy (25–27). In recent years, some progress has been made in the research and application of new therapies, such as combination therapy, development of novel antitumor targets, normalization of tumor vasculature (28), combination immunotherapy, etc., and it has been shown to greatly improve the efficacy of antitumor therapy (29–31). Analyzing the literature on the relationship between antiangiogenic therapy and TME using bibliometric methods can help researchers better understand the current development, knowledge structure, and research hotspots in this field. Here, for the first time, we conducted a bibliometric analysis of recently published articles in the above research areas, providing an important basis for their historical development and research frontiers.

In this study, publication records in the Web of Science Core Collection database were searched and analyzed from different perspectives using specialized visualization software such as VOSviewer and CiteSpace. VOSviewer is a software tool for creating and visualizing bibliometrics and web analytics for creating visual maps for co-citation analysis and co-word analysis, while CiteSpace focuses on analyzing and visualizing trends and dynamic patterns in a specific field of research, including time-series analysis and cluster analysis, among others. This study experiments with the R language in combination with both of these tools to visualize literature for descriptive statistical analysis

According to our findings, research attention on the relationship between TME and antiangiogenic therapy has been growing over the past decade, resulting in a significant increase in the number of annual publications and total article citations for antiangiogenic cancer therapies and a gradual focus of the therapies on altering TME. Of all the countries, China and the United States have contributed the most to this area of research with 2,488 and 2,230 publications, respectively. Regarding international impact and collaborative research efforts, although China, the United States, and Italy lead in publication counts as depicted in **Table 1**, the United States and Italy, despite publishing fewer papers than China, enjoy a higher frequency of citations, with Italy standing out significantly. This trend indicates that the United States and Italy wield considerable academic sway within this research domain.

Several factors might contribute to this influence. Principally, the effectiveness of international cooperation plays a role, as it broadens the global reach and impact of research by engaging a diverse network of scholars and institutions across the world. Additionally, more efficacious dissemination strategies could be at play, including presentations at esteemed international conferences, robust partnerships with renowned journals, and proactive engagement through social media and digital platforms. **Figure 3** illustrates that within the international research collaboration landscape, the United States exhibits the most robust partnership with China, with Italy following suit in collaborative frequency. It is also notable that Italy has cultivated partnerships with a broader array of countries relative to others. Consequently, these regions have established a positive feedback loop, positioning them ahead of other areas in both publication volume and citation counts.

In terms of the most productive research institutions, FUDAN UNIV ranked first, followed by SICHUAN UNIV, UNIV TEXAS MD ANDERSON CANC CTR, SUN YAT SEN UNIV, and SHANGHAI JIAO TONG UNIV, each of which produced more than 350 publications, each citing 130 or more times. In addition, CANCER RES was the most influential source of publications based on citation and co-citation analysis. Wang Y and Zhang Y were the most prolific authors with 195 publications and 291 and 230 citations, respectively. The most cited publications suggest that antiangiogenic therapy can affect TME, which lays the foundation for further development of antitumor therapy (17). Regarding the reference co-citation analysis, the themes that showed the strongest outbursts were the link between the TME and tumors and how antivascular treatment of tumors can increase therapeutic efficacy (24, 32, 33). The highest citation burst intensity (59.89) was published in CELL by Hanahan D et al., and this review article highlights the importance of the TME, showing that it consists of a wide variety of recruited, ostensibly normal cells that contribute to tumor development and marker acquisition by creating the TME. In addition to this, the article shows how anti-angiogenic therapy affects the TME to exert anti-tumor effects (17).

In addition, based on keyword co-occurrence analysis, the research trend has gradually evolved from the role of epidermal growth-induced controversy (EGF) and vascular endothelial growth factor (VEGF) in the TME to how antivascular therapy plays an important role in the tumor treatment process. In addition, nanoparticle-based drug delivery systems and immunotherapies are a topic that has been widely used in the field of antivascular therapy for tumors in the last 5 years and continues to attract the interest of researchers (34–36). Additionally, the significance of epithelial-mesenchymal transition (EMT), T-cells, and dendritic cells (DCs) is gaining increasing recognition in the field of cancer research. Tumor angiogenesis relies on blood vessels to provide oxygen and nutrients while eliminating metabolic wastes, a process that is dependent on a variety of factors in the TME such as EGF, VEGF, and so on (37). However, conventional antivascular therapy may have certain limitations in clinical application (38, 39), such as the possibility of autophagy and other pathways occurring through the TME thereby increasing the tumor’s invasive and metastatic potentials (40), in addition to the short therapeutic window, and so on. In order to address the above problems, many new therapies have emerged in recent years, such as the possibility of combining antiangiogenic drugs with conventional antitumor therapy (41); or improving drug and immune cell delivery through nanoparticle-based delivery systems to enhance therapeutic efficacy (42, 43); furthermore, recent studies have shown that antiangiogenic drugs may improve TME and thus enhance immune cell infiltration and activity, thus creating a synergistic effect with immunotherapy (44).

In summary, bibliometric analyses serve as an indispensable instrument for propelling future research and clinical practices towards more efficacious cancer treatment methodologies. They achieve this by pinpointing research trends and uncovering seminal studies that offer insights for clinical implementation. Our research underscores the enduring interest in the mechanisms through which anti-angiogenic therapies combat tumor growth by modulating the TME. Our findings reveal that the amalgamation of anti-angiogenic therapies with substrate and nanoparticle drug delivery systems, as well as immunotherapy, has emerged as a burgeoning area of interest. This approach holds promise for enhancing drug stability, improving precision targeting, and mitigating adverse effects. Furthermore, the spotlight is increasingly on the roles of EMT, T cells, and DCs. EMT is a pivotal biological process that endows tumor cells with invasive and metastatic potential. During EMT, epithelial cells undergo a transformation, shedding their original characteristics and adopting mesenchymal traits. This includes an increased capacity for cell motility and migration. The process of EMT is intricately linked to the development of tumor angiogenic mimics (VMs), which are instrumental in the tumor cells’ ability to form vascular networks. This, in turn, paves the way for tumor invasion and metastasis, presenting a novel avenue for cancer progression (45). Consequently, anti-angiogenic drugs are assuming a more prominent role in cancer therapy. For instance, the γ-secretase inhibitor DAPT can impede the metamorphosis of tumor cells into endothelial-like progenitor cells and effectively thwart the Notch signaling pathway when co-administered with bevacizumab (46), thereby curbing tumor growth. T-cells, particularly CD8+ cytotoxic T-cells, are instrumental in the tumor immune response. Dendritic cells, recognized as the most potent professional antigen-presenting cells, are capable of efficiently triggering T cell-mediated immune responses. Moreover, our bibliometric research has highlighted the synergistic potential of combining antiangiogenic therapy with immunotherapy in recent years. This combination may enhance the infiltration and functionality of immune cells by ameliorating the TME. For instance, anti-angiogenic medications can mitigate the irregularities and leakage associated with tumor blood vessels. This reduction in pressure and enhancement of blood flow within the tumor facilitates a more efficient arrival of immune cells to the tumor site (47).

In conclusion, EMT, T cells, and dendritic cells are pivotal players in tumor immunity and anti-vascular therapy. The integration of anti-angiogenic drugs with immunotherapy and nanotechnology emerges as a promising strategy to augment the efficacy of tumor treatment. Future research endeavors should delve deeper into the optimal blend and sequencing of these therapeutic modalities to optimize treatment outcomes. By merging clinical studies with bibliometric analyses, we can offer a more holistic view of the prevailing research trends and focal points within anti-vascular therapy for tumors. This approach provides essential data support for charting future research trajectories and policy development in this domain. Furthermore, it fosters interdisciplinary collaboration and dialogue, which is crucial for advancing the field of cancer treatment.

Drawing from the insights of bibliometric analysis, the domain of antiangiogenic therapy, which manipulates the TME to exert antitumor effects, has witnessed remarkable advancements. Specifically, within the pivotal biological process of angiogenesis, there has been a marked focus on the traditional EGF/VEGF signaling pathways, while also expanding research to include PDGF/PDGFR, FGF/FGFR, and TGF-β/TβR I pathways (48, 49). These explorations suggest that targeting these angiogenic pathways may pave the way for innovative therapeutic approaches to combat tumor growth and metastasis.

This analysis further indicates a pronounced trend in research evolution, characterized by a transition from examining individual angiogenic factors to embracing an integrated approach that leverages multi-target signaling pathways. Additionally, there is a burgeoning synergy between anti-angiogenic therapy and immune checkpoint inhibitors (ICIs), marking a significant stride in therapeutic strategy development (50). This evolution implies that a convergence of diverse treatment modalities could significantly remediate the TME, bolstering the capacity of immune cells to target and neutralize tumors. As our comprehension of the TME’s heterogeneity and the nuances of individual patient responses deepens, the ethos of personalized medicine is being progressively integrated into antiangiogenic therapy. Bibliometric analysis, enriched by the study of keyword co-occurrence, has traced the trajectory of research trends—from an emphasis on singular growth factors to a more holistic, multi-target treatment paradigm. These endeavors not only substantiate future research trajectories but also underpin the formulation of pertinent policies. To encapsulate, bibliometric analysis in the realm of antiangiogenic therapy affords a broad vantage point from which to scrutinize the field’s research milestones, prevailing focal points, and prospective avenues for exploration. With persistent and profound inquiry, the quest for tailored antiangiogenic treatment regimens is poised to yield more efficacious and precise clinical outcomes, instilling renewed optimism in the pursuit of cancer therapies.

Despite advances in cancer treatment with antiangiogenic therapy, a significant research effort is needed to expand its use in clinical practice. Firstly, the suitability of recruited patients to participate in cancer clinical trials based on conventional antitumor treatments such as chemotherapy should be carefully examined, not only by taking into account factors including tumor type and stage, but also by assessing synergistic effects and safety. In addition, the initial clinical dose and dosing regimen are so critical that the appropriate dose and frequency of administration should be determined based on pharmacokinetics to balance efficacy and side effects. At the same time, associated adverse events, such as hypertension, proteinuria, and bleeding, should be closely monitored (14, 51). Finally, patient-reported outcomes (PROs) should be considered as part of the assessment of treatment efficacy for quality of life and symptomatic changes (52). As evidenced by our bibliometric analysis, how antiangiogenic therapies exert antitumor effects by altering the TME has attracted long term attention from researchers, and in recent years integration with base-and-nanoparticle drug delivery systems and immunotherapies is expected to improve drug stability, enhance targeting, and reduce side effects. By combining clinical studies with bibliometric analysis, this study better demonstrates the research trends, the hot topics of tumor antivascular therapy, and so on, which not only provides data support for future research directions and related policy making in this field, but also can promote interdisciplinary cooperation and communication.

### Limitation

It is undeniable that this study has its own limitations, such as the fact that only specific databases were included in this study, leaving out literature from databases such as PubMed, in addition to the lack of diversity in the research methodology of this study, which was mainly limited to mathematical and statistical methods. In the future, we could narrow the search to different specific publications while developing software tools other than CiteSpace, VOSviewer, etc. to perform the analysis. Nonetheless, this is the first study of bibliometric analysis of the relationship between antiangiogenic therapy and the TME in cancer therapy which can help researchers to explore and develop this research area in depth.

## Conclusion

In recent decades, there has been a substantial increase in research on how antiangiogenic drugs exert their antitumor effects by affecting the TME. The current work provides the first bibliometric analysis of articles on this field, providing crucial insights and rationale for its development. Notably, China and the United States dominate this therapeutic area, and through the most cited publication, Douglas H et al. confirmed the importance of the TME, showing how new approaches such as antivascular therapy are beginning to be developed to treat cancer (17). In addition, nanoparticle-based drug delivery systems to improve drug and immune cell delivery to enhance therapeutic efficacy have also attracted long-term research interest in recent years. New anti-angiogenic therapeutic targets as well as new delivery routes may be developed in future applications in cancer research to break through existing limitations.

## Data Availability

The original contributions presented in the study are included in the article/supplementary material. Further inquiries can be directed to the corresponding author.
